# Molecular Docking of Seaweed-Derived Drug Fucoxanthin Against the Monkeypox Virus

**DOI:** 10.7759/cureus.58730

**Published:** 2024-04-22

**Authors:** Ramakrishnan Nikitha, KLG Afeeza, Vasugi Suresh, Elangovan Dilipan

**Affiliations:** 1 Physiology, Saveetha Dental College and Hospitals, Saveetha Institute of Medical and Technical Sciences, Chennai, IND; 2 Medical Physiology, Saveetha Dental College and Hospitals, Saveetha Institute of Medical and Technical Sciences, Chennai, IND

**Keywords:** monkeypox virus, methyltransferase, fucoxanthin, seaweed, molecular docking and molecular dynamic simulations (mds)

## Abstract

Background

The monkeypox virus (MPXV) is classified as a zoonotic virus of the *Poxviridae* family, resulting from the MPXV strain of the *Orthopoxvirus* genus. Seaweeds, or marine macroalgae, are abundant reservoirs of bioactive compounds that demonstrate diverse biological properties, such as antiviral actions. In the field of computational analysis, in silico analysis refers to the use of computer-based methods to study and assess biological systems and processes. To forecast the binding affinity and interaction between the discovered chemical and the target proteins of the MPXV, a molecular docking analysis was conducted.

Aim

The research aims to conduct an in silico examination of a protein-ligand interaction of a drug produced from seaweed that targets the MPXV.

Methodology

Protein Data Bank (PDB) and PubChem databases provided MPXV methyltransferase and fucoxanthin ligand compounds. AutoDockTools 1.5.7 calculated the molecular docking using the Lamarckian genetic algorithm. Autogrid created a grid box around target 8B07 active site hotspot residues. Each docked molecule's docking parameters were obtained from 100 docking experiments with a maximum of 2.5 × 10^6^ energy evaluations, a 0.02 mutation rate, and a 0.8 crossover rate. The population comprised 250 randomly selected volunteers. PyMOL was utilized to observe ligand fragment interactions.

Results

The binding energy of the ligand fucoxanthin was -5.46 kcal/mol. Fucoxanthin interacts with receptor molecules via hydrogen bonding at the amino acid level: Chain A: PHE188 and TYR189; and Chain B: LYS33, GLN37, GLY38, GLY96, ARG97, PHE115, PRO202, and SER203. The higher the negative docking score, the stronger the binding affinity between the receptor and ligand molecules, indicating that bioactive substances are more effective.

Conclusion

The findings of this study indicate that fucoxanthin, a pharmaceutical derivative generated from seaweed, had antiviral activity against the MPXV. This conclusion was reached based on protein-ligand interactions.

## Introduction

Monkeypox, a rare and possibly severe viral disease caused by an *Orthopoxvirus*, poses a substantial public health issue, especially in the regions of Central and West Africa where outbreaks are prevalent. The clinical presentation is distinguished by the presence of fever, headache, lymphadenopathy, and a unique rash [1]. The progression of monkeypox has three distinct stages: incubation, prodrome, and eruption. In countries where smallpox is prevalent, supportive care measures are used with the occasional use of smallpox medications, despite the absence of a targeted antiviral therapy [2].

The monkeypox virus (MPXV) is responsible for causing a zoonotic illness known as monkeypox. MPXV is classified under the *Poxviridae* family, *Chordopoxvirinae* subfamily, *Orthopoxvirus* genus, and MPXV species. The MPXV has a significantly high size range of 200-250 nanometers when seen by electron microscopy. Poxviruses are brick-shaped and enveloped by a lipoprotein envelope containing a linear double-stranded DNA genome. Poxviruses possess all essential replication, transcription, assembly, and egress proteins in their genome, in addition to their need for host ribosomes for mRNA translation [3,4]. The Democratic Republic of the Congo reported the first instance of human MPXV infection in 1970. The United States of America reported the first occurrence of monkeypox in a person outside of Africa in 2003 [5]. Skin-to-skin contact is a prevalent mode of transmission for the MPXV, including the use of materials or objects already contaminated by the infected individual, direct contact with the infected individual, or exposure to respiratory secretions. The transmission of the virus to the fetus may occur via the placenta of a pregnant mother. The transmission of the MPXV may also occur via direct contact with infected animals, such as through bites or scratches [6]. The MPXV gene has a homolog to COP-A44L, which encodes a segment of 346 amino acids. COP-A44L is responsible for encoding a 3-β-hydroxysteroid dehydrogenase enzyme that is necessary for the production of steroid hormones, such as sex hormones or glucocorticoids. This enzyme is responsible for converting pregnenolone into progesterone, as well as converting dehydroepiandrosterone into androstenedione. Glucocorticoids are well-recognized for their immuno-inhibitory and anti-inflammatory properties, which impact the immune system's response to the virus. Nevertheless, A44L is not essential for the process of viral replication. Thus, A44L inhibits the immune system's response and increases the production of steroids, which both influence virulence [7].

Macroscopic algae, also known as seaweeds, have been ingested for centuries in many locations in Asia and Europe. Seaweed contains bioactive chemicals. Along with the primary metabolites that are needed for proper growth, seaweeds also make a wide range of secondary metabolites in response to changing environmental factors [8]. Functional compounds derived from marine macroalgae have been used as a valuable resource, exhibiting several favorable biological properties [9]. The assessment of green, red, and brown algae revealed the existence of minerals and vitamins A, C, D, E, and B12, along with other compounds that possess fascinating characteristics. Seaweed extracts are believed to have potential use in the production of functional meals and drinks. Marine algae is the primary provider of iodine and bromine. Consuming seaweed may satisfy the body's daily need for 150 mg of iodine [10].

Fucoxanthin, a unique carotenoid mostly present in brown algae, has significant promise in the field of pharmaceuticals. Fucoxanthin is well-known for its safety profile and has attracted significant interest in clinical research due to its claimed capacity to improve metabolism without causing stimulation of the central nervous system [11]. Pharmacological studies have also shown that fucoxanthin has several effects. For example, it has been shown to lower the levels of triglycerides in the blood and liver and to control enzymes that help control cholesterol [12]. Furthermore, fucoxanthin shows potential for controlling malignancy by inducing cell cycle arrest and death and its effects on several cell functions, such as proliferation and the control of protein expression [8,13]. Fucoxanthin, a carotenoid derived from marine sources such as macroalgae and microalgae, has a diverse array of advantageous characteristics, including anti-obesity, anti-diabetic, anti-inflammatory, anti-cancer, hepatoprotective, cardiovascular, and cerebrovascular protective capabilities [14]. In order to delve further into its capabilities, it is crucial to verify its antiviral efficacy by conducting molecular docking to ascertain the ligand's binding energy with the methyltransferase receptor of the MPXV [8,15]. The efficacy of potential therapeutic interventions is significantly impacted by the gravitational attraction of a ligand, such as fucoxanthin.

Molecular docking plays a significant role in the fields of structural molecular biology and computer-assisted drug development. The objective of ligand-protein docking is to anticipate the primary binding mode(s) of a ligand with a protein that has a well-defined three-dimensional structure [16]. Molecular docking plays a crucial role in the structure-based drug design process, offering significant insights into the mechanism of action and informing the development of drug candidates that are more powerful and selective [17]. Effective docking methods employ a scoring system to rank possible dockings and examine multidimensional spaces. The use of docking in lead optimization considerably enhances the process by doing virtual screening on extensive collections of compounds, evaluating the results, and generating structural insights into how the ligands impede the target. Interpreting the results of stochastic search techniques may pose challenges, and establishing the input structures for docking is as important as the docking process itself [18]. The continuous development of computer power and algorithms has led to the evolution of molecular docking, resulting in enhanced accuracy and reliability for its use in the intricate field of drug discovery [19]. Therefore, the major objective of the study is to investigate the effectiveness of a drug derived from seaweed in combating MPXV via the interaction of proteins and ligands within the framework of the molecular docking approach.

## Materials and methods

Retrieval of protein molecule and file preparation

**Figure 1:**
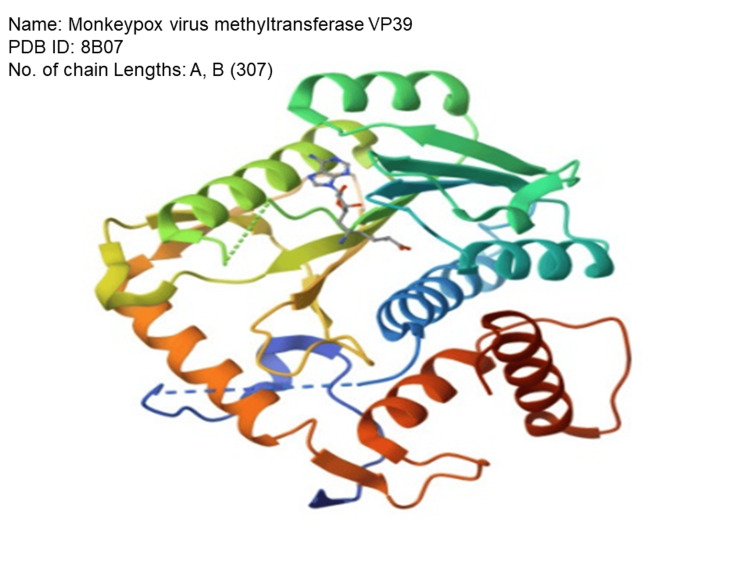
Structure of the protein molecule retrieved from the PDB database. PDB: Protein Data Bank

The three-dimensional structures of MPXV genes, namely, the methyltransferase gene (PDB ID: 8B07), were obtained from the Protein Data Bank (PDB) database and shown in Figure 1. The Discovery Studio Visualizer (visualizing tool) is used to separate the surplus water molecules and chains from the protein. The polar bonds, Kollman charges, and docking parameters in the protein structure were included using the AutoDockTools 1.5.7 software.

Retrieval of ligand and file preparation

**Figure 2:**
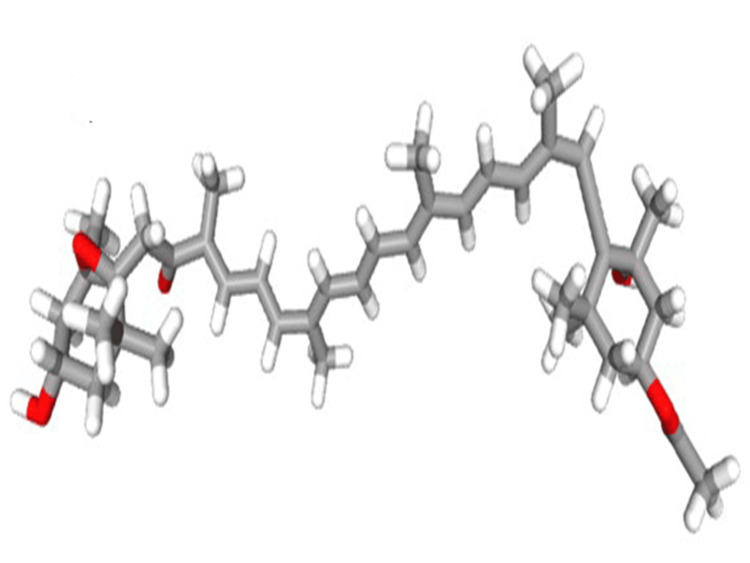
Structure of the ligand (fucoxanthin) molecule retrieved from the PubChem database.

A search was conducted in the PubChem database to extract the three-dimensional structures of the fucoxanthin molecule (PubChem ID: 5281239) (Figure 2). The AutoDockTools 1.5.7 was used to set the number of torsions (1-6), add aromaticity requirements, and set the angle cutoff (7.5). The docking parameter files were obtained using the technique that was described before.

Molecular docking

PDBQT files, docking parameter files, and grid parameter files were generated along with grid box dimensions (50×50×50). When calculating molecular docking, the Lamarckian genetic algorithm was used, and the program AutoDockTools 1.5.7 was utilized for the calculation. With the use of an autogrid, the hotspot residues that were present in the target 8B07 active site were contained inside a grid box. In order to identify the docking parameters that were subsequently applied to each docked molecule, a total of 100 separate docking experiments were conducted. These experiments had a maximum energy evaluation of 2.5 × 10^6^, and the mutation and crossover rates were measured at 0.02 and 0.8, respectively. For the purpose of keeping a record of the results, docking parameter files were produced [20,21]. A thorough examination of the binding poses of every chemical was carried out, and the conformation with the lowest energy from the most extensive cluster was chosen. PyMOL has been used to investigate the interactions that take place between the ligand fragments.

## Results

This approach is an essential component in the process of developing structure-based drugs. For the purpose of pharmaceutical composition, the interaction between the chemical and the receptor is of the utmost importance. A single cluster of conformers with a relative standard deviation tolerance of 2,000 angstroms was created by 10 docking simulations of ligands into the molecule of the methyltransferase protein. The binding energy of the ligand fucoxanthin is shown in Table 1, which shows that the binding energy is -5.46 kcal/mol. Fucoxanthin interacts with receptor molecules by forming hydrogen bonds at the amino acid level. PHE188 and TYR189 come from Chain A, whereas LYS33, GLN37, GLY38, GLY96, ARG97, PHE115, PRO202, and SER203 are found in Chain B. When the negative docking score is higher, the binding affinity between the receptor and ligand molecules is stronger. 

**Table 1:** Molecular docking result of fucoxanthin against MPXV methyltransferase domain. RMSD: root-mean-square deviation; MPXV: monkeypox virus

RMSD	Value
Binding energy	-5.46 kcal/mol
Ligand efficiency	-0.11 kcal/mol
Inhibitory constant	98.77 mM
Intermolecular energy	-10.24 kcal/mol
Total energy	-2.42 kcal/mol
Amino acid residue	Chain A: PHE188 and TYR189; Chain B: LYS33, GLN37, GLY38, GLY96, ARG97, PHE115, PRO202, and SER203

**Figure 3:**
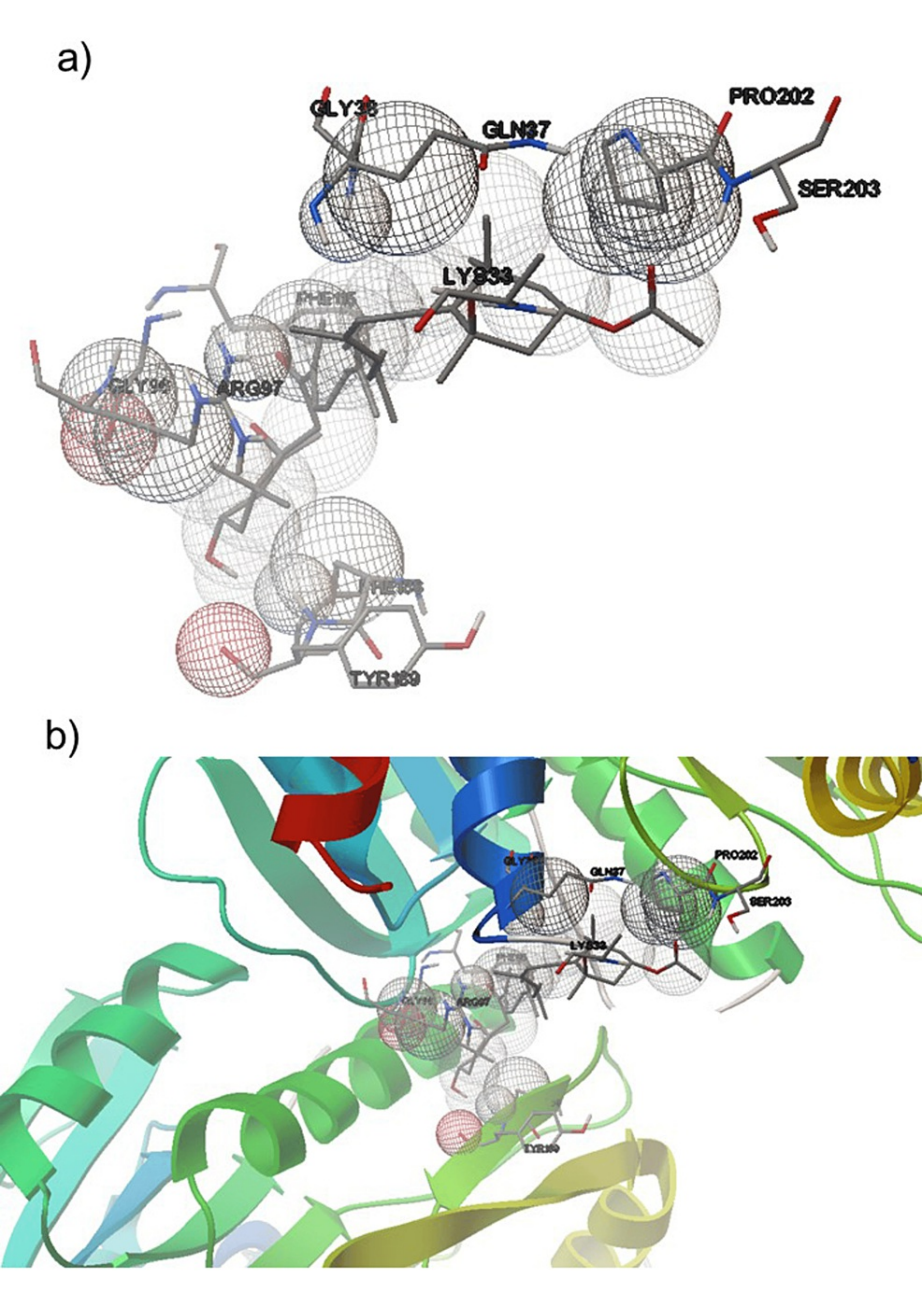
Molecular docking of MPXV methyltransferase receptor with fucoxanthin. The receptor interaction with the ligand is represented by a blue color. a) Protein-ligand interaction. b) Ligand molecule docking with a chain of MPXV methyltransferase. MPXV: monkeypox virus

The formation of hydrogen bonds was the primary method by which fucoxanthin interacted with the molecules that make up receptors. Certain amino acid residues inside the receptor are responsible for the formation of these chemical bonds. Chain A and Chain B, two distinct protein chains, include the amino acid residues involved in the binding process. Chain A has PHE188 and TYR189, while Chain B contains LYS33, GLN37, GLY38, GLY96, ARG97, PHE115, PRO202, and SER203 (Figure 3).

## Discussion

Molecular docking is a crucial and advanced approach in computational chemistry for drug development. It involves analyzing the interactions between targeted ligands and the active region of a protein's receptor [22]. In this study, the molecular docking analysis revealed that fucoxanthin, a naturally occurring chemical found in several types of seaweed, had a significant binding energy of -5.46 kcal/mol [23,24]. Based on the docking analysis conducted by Arasu et al. [25], it was shown that many ligands have a binding affinity for the MPXV viral protein, ranging from -5.0 to -6.7 kcal/mol. Viral surface proteins can bind to the surface receptor of the target cell, making them very promising for the production of vaccines [26]. In vitro, several natural biomolecules possess the capacity to impede the replication machinery of a specific virus type, hence preventing infection. The ligand's optimal posture was determined based on the maximal binding energy value (kcal/mol), which considers all potential beneficial interactions with residues in the protein's active region [27]. Binding energy is a critical determinant in molecular docking experiments. The measurement quantifies the strength of the interaction between a ligand (fucoxanthin) and a receptor (chemicals associated with the MPXV) [28]. The presence of a higher negative binding energy suggests a greater affinity between the ligand and receptor, so suggesting that bioactive compounds such as fucoxanthin may exhibit enhanced efficacy in inhibiting the MPXV [20,21]. The primary mode of interaction between fucoxanthin and receptor molecules involves the formation of hydrogen bonds. These connections occur at certain amino acid residues within the receptor. Sahoo et al. [29] performed a computational drug repurposing investigation to identify existing, authorized drugs that might potentially inhibit the crucial MPXV proteins, thymidylate kinase, and D9 decapping enzyme, by using the analogous protein structures found in the vaccinia virus. According to the study findings, it has been shown that Chain A and Chain B, which are two separate protein chains, include a set of amino acid residues that play a crucial role in the process of binding. Chain A consists of PHE188 and TYR189, whereas Chain B comprises LYS33, GLN37, GLY38, GLY96, ARG97, PHE115, PRO202, and SER203. Similarly, the study discovered that the mitoxantrone (MXN) standard was associated with both hydrophobic and hydrogen bonding interactions with the thymidylate kinase amino acid residues Ser15(B), Arg93(B), Asp13(B), Glu142(B), Asn37(B), and Thr18(B) with bond distances of 3.34 Å, 2.80 Å, 3.08 Å, 2.78 Å, 2.98 Å, and 2.99 Å, respectively. Tyr35(B) was the only amino acid residue that demonstrated hydrogen bonding interaction with MXN, with a bond distance of 3.09 Å. The hydrophobic interactions with MXN included Lys14(B), Tyr144(B), Glu145(B), Leu53(B), Arg41(B), Lys17(B), and Gly16(B) [30].

The participation of these amino acids in the hydrogen bonding mechanism with fucoxanthin indicates a very specific and directed interaction. Accurate interactions play a crucial role in the advancement of potential pharmaceuticals as they ensure the effective binding of the ligand to the specific site on the receptor molecules associated with viruses [31]. Moreover, the significance of a lower docking score cannot be overstated. The docking score of -5.46 kcal/mol indicates a strong binding affinity between fucoxanthin and the receptor molecules associated with the MPXV. This implies that fucoxanthin has promising potential as a powerful inhibitor of the MPXV, making it a compelling subject for further investigation and advancement as a medicinal agent derived from seaweed [32-34].

Limitation

Docking studies may have limited effectiveness in capturing the conformational changes that proteins experience during ligand interactions. In real-world circumstances, the intrinsic diversity of protein-ligand interactions may provide results that diverge from the predictions generated by docking simulations. Experimental validation is necessary because of the predictive nature of molecular docking findings. The efficacy of fucoxanthin in treating MPXV is still unknown since it has been studied in vitro (cell culture) and in vivo (animal model). Furthermore, computational studies may neglect the cellular environment, which is critical to the actual interaction of a medication and its target.

## Conclusions

The current investigation confirms that fucoxanthin has promise as a bioactive compound for the treatment of monkeypox. This conclusion is derived from an analysis of its binding energy and its interactions with certain amino acid residues within the receptor molecules linked to the MPXV. The greater negative docking score, which indicates a strong binding affinity, has generated confidence in the potential of seaweed-derived chemicals to produce effective treatments for battling this infectious sickness. Additionally, it will be important to investigate the therapeutic efficacy of fucoxanthin against the MPXV via in vitro (cell culture) and in vivo (animal model) investigations. 
